# School-based social skills group training (SKOLKONTAKT™): a pilot randomized controlled trial

**DOI:** 10.3389/fpsyg.2023.1128288

**Published:** 2023-07-27

**Authors:** Anna Fridell, Christina Coco, Anna Borg, Sven Bölte

**Affiliations:** ^1^Center of Neurodevelopmental Disorders (KIND), Centre for Psychiatry Research, Department of Women’s and Children’s Health, Karolinska Institutet, Stockholm Health Care Services, Region Stockholm, Stockholm, Sweden; ^2^Child and Adolescent Psychiatry, Stockholm Health Care Services, Region Stockholm, Stockholm, Sweden; ^3^Curtin Autism Research Group, Curtin School of Allied Health, Curtin University, Perth, WA, Australia

**Keywords:** autism, social skills, group-based intervention, school-based intervention, goal attainment scaling, quality of life

## Abstract

**Purpose:**

Differences in socio-communicative behaviors contribute to social challenges for autistic learners at school and, in turn, are associated with increased risks of educational underachievement, social exclusion, and mental health issues. Given that intervention delivery in natural contexts may enhance skills generalization, build support capacities in society, and have practical advantages for youth and families, SKOLKONTAKT™ has been adapted from the clinically based social skills group training KONTAKT™ for mainstream educational settings to mitigate these risks.

**Methods:**

A pilot, randomized controlled trial with active controls was conducted in a mainstream Swedish high school. Autistic learners and students with social skills challenges (*N* = 33; *M*_AGE_ = 17.5) were randomized to SKOLKONTAKT™ (*n* = 17) or active control (*n* = 16). Efficacy was measured at post and follow-up (3 months) on social skills [Social Skills Group Assessment Questionnaire (SSGQ); primary outcome] by parent-, self-, and (masked) teacher-report as well as self-reported life quality and social goal attainment.

**Results:**

Despite COVID-19 challenges, 70.6% (*n* = 12) completed SKOLKONTAKT™, and 87.5% (*n* = 14) completed control groups. SKOLKONTAKT™ improved on a series of items on SSGQ as well as subjective life quality beyond controls. A larger proportion of social goals were attained, and side-effects were of little impact and proportionally fewer in SKOLKONTAKT™.

**Conclusion:**

SKOLKONTAKT™ is a safe, feasible, and promising intervention option for autistic learners in mainstream educational settings. A larger-scale study is desirable to confirm the effects identified in this pilot study.

## Introduction

Differences in patterns of social communication and interaction and associated challenges with complying with mainstream scholastic demands and expectations are hallmarks of autism spectrum conditions (henceforth autism) (ICD-11; World Health Organization, 2019/2021). Understanding autism as a neurodivergent condition and the extreme end of a trait (Constantino and Charman, 2016; Pellicano et al., 2018) has caused many countries to mandate inclusive education for autistic students (e.g., Roleska et al., 2018; van Kessel et al., 2019). Yet, autistic learners are at higher risk of social exclusion and adverse outcomes in school settings. Notably, students with social challenges specifically rarely reach their full academic potential (Domitrovich et al., 2017), which may contribute to negative long-term vocational outcomes (Clarke et al., 2021). School-aged neurodivergent learners are also more likely to experience loneliness and victimization (Locke et al., 2010; Zeedyk et al., 2014), which may contribute to mental health issues and lower quality of life (Demir et al., 2012) as well as school absenteeism (Anderson, 2020). Successful inclusive school settings require accommodation of learning environments for neurodivergent students (Kim et al., 2018; Pellicano et al., 2018), yet research indicates that regular school staff are not well prepared to practice inclusion of neurodivergent students (Bölte et al., 2021b; Leifler et al., 2022a). In addition, autistic learners may not incidentally acquire or develop socio-communicative understanding, skills, or strategies over time by mere proximity to non-autistic peers. Given this mismatch, environmentally and individually, targeted support strategies are crucial to combat risks of adverse outcomes for these students.

Social skills training aims to expand the socio-communicative understanding and behavioral repertoire through a variety of approaches and strategies, including behavioral skills training, social stories, antecedent interventions and reinforcement, peer mediation, and video modeling (Radley et al., 2020). It may positively affect social behaviors in adolescent autistic learners with average cognitive abilities (McKeithan and Sabornie, 2020), autistic learners with co-occurring complex communication needs and intellectual disability (Babb et al., 2021), and autistic learners in inclusive school settings (Dean and Chang, 2021). Group-based social skills training formats may offer added benefits including real-time practice of taught skills with peers in a structured environment (Tachibana et al., 2018) and can serve more students with better cost-effectiveness (Myhr and Payne, 2006; Tucker and Oei, 2007) for some autistic learners.

Though scarce in educational settings, in clinical settings, manualized, group-based social skills training (c-SSGT) is one of the most widely applied and evaluated psychosocial interventions for school-aged verbal autistic children and adolescents in the average range of intellectual functioning (Hall et al., 2018). Here, a comprehensive set of evidence-based strategies for autistic youth (Hume et al., 2021), such as modeling, behavioral practice, and direct instruction, are often integral parts of a peer-group setting with one or several group leaders. In a meta-analysis of 18 randomized control trials comprising 745 participants, c-SSGT showed an overall positive medium-sized effect (*g* = 0.51) on social competence outcomes (Gates et al., 2017). The hitherto largest randomized controlled trial in autism compared c-SSGT KONTAKT™ to waitlisted controls in regular clinical settings (Choque Olsson et al., 2017). Manualized, 12 (short) or 24 (long) weekly sessions include group discussions and practical social activities—implemented through learning and cognitive behavioral principles—with children (8–12 years) or adolescents (13–18 years). Socio-communicative competence improved primarily for girls (*d* = 0.33) and adolescents (*d* = 0.40) following short KONTAKT™ (Choque Olsson et al., 2017) while large effects were found for children and adolescents (d = 0.82) following long KONTAKT™ (Jonsson et al., 2019).

Reviews of SSGT stress the importance of considering SSGT in naturalistic school environments (Gates et al., 2017). Benefits may include added flexibility by which support may be accessed as well as possible spin-off effects in terms of the qualifications of the school-staff (Leifler et al., 2022b). Moreover, while c-SSGT manuals typically include activities aiming to promote generalizability, e.g., homework assignments, parent participation, and social excursions outside the clinic (Choque Olsson et al., 2017; Jonsson et al., 2019), the main intervention setting is detached from the regular, everyday environment. School-based SSGT (s-SSGT) may further facilitate the generalizability of training outcomes beyond the intervention setting by reducing demands on skills transfer, which may be particularly challenging for autistic learners (Neely et al., 2016). Indeed, adapting c-SSGT to s-SSGT is feasible and may improve teacher-rated socio-communicative function, family frequency ratings of hosted and invited get-togethers with peers, and adolescent-rated social competence (Laugeson et al., 2014; Dean et al., 2020).

Notably, however, qualitative studies indicate that even participants with little gains in terms of standardized socio-communicative measures experience socially relevant and meaningful outcomes (Choque Olsson et al., 2016; Afsharnejad et al., 2022). Few standardized scales are designed to assess defined natural contexts where social skills are performed (i.e., generalization). Moreover, by definition, standardized measures are not able to consider individual priorities and personally important areas of development. Goal Attainment Scaling (GAS; Kiresuk et al., 1994) is a goal-setting procedure that has been applied in a variety of care and support settings. As an added component in SKOLKONTAKT™, it has the potential to address the individual variability and priorities of autistic learners (Bishop et al., 2016) as well as define generalizability to important naturalistic contexts while detailing outcomes at the group level. GAS has been shown to be a feasible and safe measure in school-aged autistic learners (Ruble et al., 2012, 2022) and as a goal-setting procedure, and it may motivate participants to engage with the intervention (Locke and Latham, 2019).

Given the potential of SSGT in a school setting, our research center has adapted c-SSGT KONTAKT™ to ‘SKOLKONTAKT™’ (Eng. ‘SCHOOL-KONTAKT): an s-SSGT aimed at students with formally documented social communication and interaction challenges at school and following several principles. First, while studies investigating the effects of social skills training at school have often applied research staff as intervention providers (Dean and Chang, 2021), using qualified school staff may be crucial in terms of contextualizing the training. Staff-led training is consistent with regular schooling—for the student as well as for the sustainability of the intervention (Watkins et al., 2017). Second, intervention development was conducted with the aim to enhance stakeholder acceptability of intervention procedures, i.e., the social validity, to reduce barriers for effective implementation (Fleury et al., 2014). Findings from our previous qualitative multi-perspective interview study have informed the social validity of SKOLKONTAKT™ compared with other socially engaging group activities at school (Leifler et al., 2022b). Given adequate support and resource planning, school staff considered SKOLKONTAKT™ as a well-placed and motivational intervention for their school. Participating youths, school leaders, and SKOLKONTAKT™ facilitators experienced an improved social climate and culture at school as well as social behavior development among students, such as knowing how and daring to engage in small talk with peers, making one’s voice heard, and managing social situations. Little to no such experiences were reported for other socially engaging group activities.

The present study aims to add quantitative data to these findings to investigate acceptability, feasibility, and preliminary efficacy outcomes of manualized SKOLKONTAKT™ in comparison to non-specific effects of engaging in social group activities with peers for autistic learners and students with formally documented socio-communicative needs. Moreover, understanding of the extent of skill transfer beyond the intervention (i.e., generalization) was examined by use of goal attainment outcomes.

## Materials and methods

### Study design

Following approval by the Swedish Ethical Review Authority and study protocol registration on Clinical Trials (ID: NCT04302818), a pragmatic randomized, controlled pilot study evaluated the feasibility and preliminary effectiveness of SKOLKONTAKT™ in comparison to social activity control groups in a community setting. Participants were randomized (1:1 to each arm of the study, stratified by gender) using an online program.^1^ Students enrolled in active control groups were invited to SKOLKONTAKT™ training following study participation. Participant, caregiver, and teacher perspectives were collected pre-and post-intervention as well as at follow-up 3 months after the intervention. In addition to feasibility, defined as training attendance and completion, the primary outcome was defined as changes in social skills according to the Social Skills Group Assessment Questionnaire. Secondary outcomes included quality of life, achievement of personally meaningful goals, and negative side effects. The study applied partial, single-masking procedures. Student mentors or teachers, not part of the study, were recruited to provide presumptive blinded baseline, outcome, and follow-up data on the primary outcome of the study (Social Skills Group Assessment Questionnaire). A member of the research team remained blinded for group allocation following randomization to assess formulation and outcome on personally, meaningful goals (Goal Attainment Scaling). Following staff availability, implementation of SKOLKONTAKT™ and social activity control groups was conducted by school staff in a crossover design, e.g., leading an active control group following training of a SKOLKONTAKT™ group. Protocol fidelity was encouraged by structured questions during the supervision meetings and direct observation of several SKOLKONTAKT™ sessions over the course of the study.

### Participants and recruitment

Students were identified, assessed, and recruited purposefully by school staff in collaboration with local student health services. Eligible students were either in the average psychometrically defined intellectual range of functioning (IQ ≥ 70 +/− 5) or had no clinical or educational indication of intellectual disability, had sufficient Swedish language proficiency, explicit motivation by own verbal account, and had formally documented social communication and interaction challenges as indicated by individual action plans (IAPs) or neurodevelopmental condition diagnoses. School staff were advised that students with current psychiatric conditions (e.g., moderate to severe depression) or severe externalizing behaviors (e.g., severe oppositional defiant disorder) were not eligible for participation.

In total, 33 adolescent and young adult students were enrolled in the study, of which 17 students were randomized to SKOLKONTAKT™. According to school or medical records, full-scale IQ ranged between 68 and 114 (*M* = 91.23; SD = 12.14) (33–42% missing IQ data). The total sample of enrolled students comprised of 17 girls and 15 boys (self-identified; missing gender information for 1 student), aged 16–21 years (*M* = 17.5, SD = 1.33). Overall, 7 students were diagnosed with autism (21%), 13 students were diagnosed with autism and ADHD (39%), 5 students were diagnosed with ADHD (15%), and 7 students (21%) were diagnosed with either other psychiatric and neurodevelopmental conditions (e.g., dyslexia) or had subclinical social challenges at school (diagnostic information for 1 participant missing).

Enrolled students without a formal autism diagnosis (*n* = 13) included 8 girls with clinical diagnoses of ADHD (*n* = 4), other psychiatric or neurodevelopmental disorders than ADHD or autism (e.g., dyslexia) (*n* = 4), or subclinical social challenges at school (*n* = 4). Diagnostic information for one student was not available for the study, and they were categorized as non-autistic. Non-autistic learners had average full-scale IQs (*M* = 99.8; range = 92–108), though data were missing for 6 non-autistic participants. All were enrolled in the training owing to existing individual support action plans detailing social skills challenges at school. Sample characteristics are shown in Table 1.

**Table 1:** Sample characteristics.

	SKOLKONTAKT™	Social activity control	Total	Dropouts
Number	17	16	33	7 (SKOLKONTAKT = 5)
Gender	(9 girls; 8 boys)	(8 girls; 7 boys) (1 missing)	(17 girls; 15 boys)	(3 boys; 3 girls) (1 missing)
Age (year)	17.9 (16–21)	17.1 (16–20)	17.5 (16–21)	17.3 (15–19)
IQ full scale (WISC-IV)	94.11 (80–114)	89.23 (68–109)	91.23 (68–114) (33% missing data)	**
IQ verbal (WISC-IV)	106.67 (87–136)	98.,15 (81–112)	101.64 (81–136) (33% missing data)	**
IQ perception (WISC-IV)	98.75 (88–112)	97.64 (67–112)	98.11 (67–112) (42% missing data)	**
Only autism	1	3	4	0
Only ADHD	1	2	3	1
Only other	1	2	3	1
Autism + ADHD	2	3	5	1
Autism + Other	2	1	3	0
ADHD + Other	2	0	2	1
Autism + ADHD + other	5	3	8	1
Subclinical social challenges*	3	1	4	2
No information on Clinical diagnosis	0	1	1	0

Other = other psychiatric clinical diagnosis and neurodevelopmental conditions (e.g., dyslexia). *Three students indicated ongoing neuropsychiatric assessments, though this could not be confirmed in the present study. **These data are not reported as more than 70% of the data is missing.

### Setting and procedure

SKOLKONTAKT™ was piloted in a mainstream senior high school in central Stockholm, Sweden. Students follow the national curriculum objectives, but the school allocates extra resources for students in the form of targeted individual support, classes of reduced size, and higher school staff-to-student ratios. Additionally, for all students, IAPs are created and followed up by a designated mentor who is part of the school staff. These were used as formal documentation of the need for social support during the study period. Between the fall of 2019 and fall of 2020, SKOLKONTAKT™ and social activity groups were run once per spring and fall term, respectively. Intervention groups were conducted in parallel to three school terms—cycle 1 during fall 2019, cycle 2 during spring 2020, and cycle 3 during fall 2020. No sessions occurred during school breaks. Sessions occurred during school hours but were not part of the regular school schedule. Three female and three male trained and monthly supervised group facilitators were teachers (*n* = 4) and school counselors (*n* = 2) with extensive professional experience working with children with neurodevelopmental conditions and social challenges (range = 11–25 years). Their average age was 52 years (range = 39–61), and all had university degrees and worked full-time at the participating school at the time of the study. Early in the school term school staff informed students and their caregivers about the possibility of study participation in the coming intervention groups. Interested families were later invited to an information meeting with a member of the research team where they were informed of the intervention and the study protocol prior to randomization. Each participant formulated personal, meaningful, and measurable goals relating to social skills at school with support from a group facilitator in an individual session (45–60 min) prior to intervention start. An overall goal was divided into five steps of goal-attainment. Each step was given a numeric representation according to the GAS scoring system (i.e., −2 to +2; Ruble et al., 2012). The final goal was also rated in terms of subjective level of difficulty. Group facilitators followed up goal attainment individually with each participant when the intervention was finalized each term. School and session attendance was reported by the school administration or manually by group facilitators following each term. Unwanted side effects were monitored and discussed in supervision throughout the study.

#### SKOLKONTAKT™ intervention

Adaptation of KONTAKT (Bölte, 2018) was led by a working group at KIND, targeting primarily adolescent students at this stage (for details on the KONTAKT intervention, please refer to Choque Olsson et al., 2017). Following an iterative, multi-professional Delphi process (Boberg and Morris-Khoo, 1992), including school staff, the adapted format was included in a clinical/educational development project in a school in a municipality in central Sweden, including a sample of seven neurodivergent adolescent students. Follow-up stakeholder interviews resulted in the finalization of the intervention format for the current pilot study. SKOLKONTAKT™ largely reflects the 12-session (short) version of the KONTAKT™-protocol (Bölte, 2018), maintaining its core principles and content. A manualized intervention, SKOLKONTAKT™ (Coco et al., 2023) is guided by activity books participants, their guardians, and school staff (i.e., group facilitators) respectively. Groups (4–8 students), guided by 2–3 facilitators with professional experience of supporting autistic learners, meet over 12 weeks. Teaching formats include didactic instruction, discussion, and problem-solving as well as practice. Structured social activities such as theme-based discussions and group exercises such as role-play, emotion recognition training, and other activities targeting general non-verbal and verbal cooperation skills make up sessions. Group facilitators enhance learning by underlying principles related to established learning principles and evidence-based strategies for social skills training, including behavioral activation, rehearsal, reinforcement, functional analysis, and psychoeducation.

Compared to KONTAKT™, in SKOLKONTAKT™ participants receive training in their natural educational environment, and training is delivered by regular school staff, not clinicians or researchers. Facilitators receive methodological training and regular supervision (1/month) during interventions (clinicians experienced in neurodivergence and cognitive behavior therapy). Moreover, a wider participant criterion was set, including increasing the upper age range (max. 20 years) considering the inclusion of students with prolonged schooling as well as substituting a clinical diagnosis of autism with “demonstration of autism-like social challenges with significant impact on academic performance based on IAPs at school with s-SSGT as a reasonable support strategy.” The latter was motivated by (i) the variability of needs within the neurodivergent student population, thus requiring variable support, and (ii) the presence of autism-like social challenges regardless of clinically established autism diagnostic status. In Sweden, decisions on needs for targeted support are documented in the IAPs—these are mandatory for students with challenges interfering with academic performance in any school format and are regulated by laws and provisions, including the Swedish Education Act (Utbildningsdepartementet SFS, 2010). Further, SKOLKONTAKT™ includes shorter sessions (50 min), increased session frequency (three times weekly), weekly missions conducted in-session, more school-related theme-based discussions, and informing guardians (as well as other relevant school staff) through weekly letters (activity book) rather than including them in sessions to adhere to change of setting (from clinic to school-grounds) and group facilitator role (from psychologist to school staff). See Supplementary Tables S1, S2 in Supplementary materials for details on SKOLKONTAKT™.

#### Social activity control group

SKOLKONTAKT™ was compared with social activity control groups with group meetings occurring in parallel to SKOLKONTAKT™ (three sessions/week of 50 min each over 12 weeks) with 4–8 participants and 2–3 group facilitators. To control for unspecific effects of recurring, structured social activities with peers, a schedule of social group-activities was created for the study. Weekly sessions include baking, a physical activity, as well as boardgames according to a schedule over 12 weeks. Active control groups did formulate individualized, social, and school-related goals but did not have weekly missions or other structured goal-related work.

Following the COVID-19 outbreak early in 2020, Swedish high schools were generally required to provide remote teaching to students. While this mandate was temporarily removed as the spread of the virus reduced during the summer of 2020, hybrid alternatives remained in many high schools (Folkhälsomyndigheten, 2021). Specifically for the present study, regular on-site mandates were implemented for group cycle 1 (2019), new mandates were implemented during group cycle 2 (2020), and hybrid alternatives were available for group cycle 3 (2020). Overall, the pandemic entailed similar changes to both group conditions including remote delivery. Both groups conducted real time group sessions online via videoconferencing systems. Social activity control groups were conducted as intended, albeit remotely. Changes to SKOLKONTAKT™ sessions included adaptation of group exercises as well as individual coaching of weekly missions (rather than during group sessions). The latter implicated shortening of session lengths by 10 min during which participants had individual contact with facilitators. Weekly missions were conducted individually and remotely when sessions were conducted remotely (no similar adaptation was made in social activity control groups as they did not have weekly missions). Thus, equivalence in the extent of support provided in face-to-face and remote SKOLKONTAKT™ is assumed. Moreover, to facilitate remote intervention delivery, activity books for facilitators were adapted and provided for school staff, and additional materials were created to be used online. All participants were informed of these changes verbally and in writing and were requested to provide updated informed consent to continue in either group.

### Measurements

Due to the significantly increasing COVID-related pressures of the school organization, some secondary measures were dropped prior to the third cycle of groups (hybrid-intervention delivery) retaining only SSGQ and GAS for all three cycles. KIDSCREEN-27 pertains only to cycles 1 and 2. Pre-intervention parent-rated SRS was included to investigate SSGQ validity.

#### Attendance and completion

Attendance to SKOLKONTAKT™ and activity control sessions and completion was registered and calculated by proportion (in %) of actual attendance out of expected attendance, i.e., all sessions. While students follow the national high school curriculum, individual students’ education planning adhered to IAPs—often including prolonged schooling and individualized weekly lesson schedules, wherefore proportion of actual attendance (reported in %) out of expected attendance for the duration of the intervention was calculated for school attendance.

#### Social Skills Group Assessment Questionnaire

A Swedish translation of the Social Skills Group Assessment Questionnaire (SSGQ) (Goldstein and Pollock, 1988; translated by authors CC and SB) was applied as the primary outcome to assess intervention-related changes in social communication skills. The SSGQ is a self-and informant-administered rating scale developed for clinical use and includes 23 items stating pragmatic, everyday social skills including “Beginning a conversation,” “Working cooperatively,” “Apologizing,” and “Asking questions appropriately.” Skills are evaluated in relation to impressions of others’ social behaviors by grading ability ranging from 1 or 2 (“is very poor at this skill”), 3 or 4 (“exhibits this skill as well as others”), to 5 or 6 (“exhibits this skill better than others”). The completion time is about 15 min. Outcomes are analyzed based on the total score (score range: 23–138), outcome per reporter, as well as the 24 individual items outcome scores. The SSGQ is a face and content valid measure specifically operationalizing the socio-communicative behavioral targets of SKOLKONTAKT™. Internal consistency for SSGQ in the study was excellent across raters with a Cronbach’s Alpha for the total of *r* = 0.95 for self-, *r* = 0.94 for parent-, and *r* = 0.97 for teacher ratings (*p* < 0.001). The parent-rated Social Responsiveness Scale 2^nd^ edition (SRS; Constantino and Gruber, 2019) was used to assess the convergent validity of the instrument. Validity of the SSGQ with the parent-rated Social Responsiveness Scale 2nd edition was excellent at baseline (*r* = −0.81, *p* < 0.001).

#### KIDSCREEN-27

We collected the short version of the KIDSCREEN (Ravens-Sieberer et al., 2007) as secondary outcome to collect self-reported changes in quality of life. It contains 27 Likert-style items covering five subscales including Physical Well-Being, Psychological Well-Being, Autonomy and Parents, Peers and Social Support, and School Environment. The instrument has demonstrated satisfactory internal consistency with a Cronbach’s Alpha *r* > 0.70 for all subscales. In the present study, item responses are numerically translated indicating the range 1 = poorer life quality to 5 = better life quality. Completion time is about 10–15 min.

#### Goal Attainment Scaling

Goal Attainment Scaling (GAS) (Kiresuk et al., 1994) was used to evaluate progress toward personally meaningful goals. Goal construction followed general instructions outlined in Ruble et al. (2022) whereby (i) an overall measurable goal with relevance relating to social skills at school was formulated, (ii) progress was detailed in five ordinal numerically and qualitatively described levels ranging performance ability towards the goal (−2 to +2) with much less than expected outcome (−2) indicating baseline performance ability (i.e., no progress towards goal, e.g., Schlosser, 2004), −1 (somewhat less than expected), 0 (expected level of outcome, i.e., goal-attainment), +1 (somewhat exceeding goal), and +2 (significantly exceeding goal) (Kiresuk et al., 1994), and (iii) assessment of progress was conducted at post-intervention (Lee et al., 2022). While this five-point scale allows for aggregation of outcomes at the group level, formulated goals need to be of sufficient quality for effective scaling and result presentation at the group level (see, e.g., Krasny-Pacini et al., 2016). There is some debate relating to the psychometric properties, and adequacy of transforming GAS-scores to standardized scores, and including them for inferential, statistical analysis (e.g., Schlosser, 2004; Krasny-Pacini et al., 2016). In the present study, several procedures were conducted to minimize potential bias in goal-setting procedures (i.e., scale construction and measurement accuracy): (i) training and supervision (part of core methodological training) as well as written instructions and checklists for facilitators, (ii) pre-intervention blinded ratings of goal quality, (iii) pre-intervention subjective ratings of level of difficulty (0–5; 5 = very difficult) by each student, (iv) post-intervention blinded assessment of outcome score as well as group leader report of retrospective baseline performance ability (0–3; 1 = could perform more or less independently, 2 = could perform given some support; 3 = could not perform independently or with support), and (v) development of a list of main goals and corresponding GAS formats (*n* = 34; “GAS-catalogue”) by joint formulation [AF, CC, AB], separate formulation according to GAS format followed by cross quality rating [split in half by authors AF and CC], and subsequent final review by author [SB].

Specifically, goal quality at pre-intervention (baseline formulations) as well as for goals developed for the GAS catalogue was rated according to recommended criteria (by Ruble et al., 2012, Krasny-Pacini et al., 2016) whereby goals use objectively measurable criteria for change towards the overall goal (‘measurability’). This entails only one behavioral dimension of the target goal, e.g., not both eating and exercise towards a health-related overall goal (‘unidimensionality’), using scaling levels that build upon previous levels (excluding level baseline at-2) such that previous behaviors are clearly attained prior to progressing to the following (‘overlap), and that goals are relevant according to the intervention focus, i.e., school and social skills (‘relevance’). Goals were rated based on each qualitative criterion on an ordinal scale (1 = aspect not fulfilled; 2 = aspect partially fulfilled; 3 = aspect fulfilled) with written instructions by a researcher unaware of the participant’s group allocation. Final quality criteria ratings (mean; range) for goals included in the goal catalogue were Equidistance (*M* = 2.4; range = 2–3), Measurability (*M* = 2.9; range = 2–3), Unidimensionality (*M* = 3; range = 3), Overlap (*M* = 2.9; range = 2–3), and Relevance (*M* = 2.9; range = 2–3). The GAS goal catalogue was available for group cycles 2 and 3. Goal-attainment results are presented descriptively.

#### Negative Effects Questionnaire

An adapted version of the 32 item NEQ the questionnaire (Rozental et al., 2016, 2019) was used to systematically assess side-effects. The words “treatment” and “therapist” were changed to more context-relevant wordings, i.e., “intervention” and “group trainer” (i.e., group facilitator). Items comprised six factors: symptoms (e.g., “I felt more worried”), quality of treatment (e.g., “I did not always understand my treatment”), treatment dependency (e.g., “I think that I have developed a dependency on my treatment”), stigma, (e.g., “I became afraid that other people would find out about my treatment”), hopelessness (e.g., “I started thinking that the issue I was seeking help for could not be made any better”), and failure (e.g., “I lost faith in myself”). The student indicates the presence or absence of a given side effect. In case of presence, the intensity (or impact) of the item is indicated on a Likert-style scale ranging “not at all” (0), “slightly” (1), moderately” (2), “very” (3), and “extremely” (4). Finally, the students report if this side effect is a consequence of the intervention or other circumstances. The present study reports side effects attributed to interventions only.

### Analysis and statistics

Outcome measures were analyzed according to the intention-to-treat principle with missing variables included by last observation carried forward if a maximum of 10% of datapoints are missing. Included for analysis are participants with pre-and post-data with less than 10% missing per measure. Thus, participants who dropped out were not included in the analyses. All analyses were computed with IBM SPSS 27.

A series of mixed-measures analyses of variance were conducted with time (pre-, post-, and follow-up timepoints) as a within-subjects factor and group (SKOLKONTAKT™ vs. social activity control) as a between-subjects factor. Computed scores include the total raw score of the primary outcome SSGQ per informant (student, parent, and teacher ratings), the total and subscale raw scores of the secondary outcome KIDSCREEN-27, as well as the total school and intervention session attendance rates, respectively, pre-to post-intervention. Furthermore, separate Pearson correlations were computed to estimate the association between school and training attendance per intervention group.

Thereafter, independent t-tests were run at the item level (pre-to follow-up raw score change) of the SSGQ for participant, parent, and teacher ratings. Item analysis for the primary outcome was deemed informative to investigate potential specific effects of SKOLKONTAKT™ on single social skills that are targeted during the training (e.g., start a conversation; ask for help). Due to the exploratory nature of the pilot study with its focus on feasibility and limited sample size, we applied an uncorrected alpha level of *p* = 0.05 for all inference statistics. Thus, we tolerated an increased risks for type 1 errors in favor of reducing risk for type 2. Pandemic-related restriction pertained to all students enrolled in the present study. For analyses they were therefore considered a common change for both treatment and control conditions and will not be corrected for.

Attendance and completion as well as the secondary outcomes of goal-attainment (GAS) and side effects (NEQ) were analyzed descriptively. Outcome progress towards personally meaningful goals according to GAS was calculated by constructing a summary outcome score in the post-assessment interview based on the most credible source of information (i.e., the student, the group facilitator, or observation by other school staff). Source credibility is based on ability to motivate the outcome, e.g., by having observed social skills at post-interview. The proportion of those deemed the most credible sources is reported. Owing to individual variability in how many GASs were formulated (ranging 1–3), outcome scores of goals with the most or second-most progress were included in analyses. Additionally, reflexive thematic analyses (Braun and Clarke, 2019) were conducted on all goals included in quantitative analyses aiming to elucidate chosen goal themes. Qualitative analysis was conducted based on the titles of goals by author AF. If it was necessary to inform goal content, scaling levels were investigated. Goals were categorized into themes on the semantic level using an inductive approach. Final thematic categories were reviewed by a senior author (SB). Minimization of bias in scale construction and measurement by quality rating criteria is calculated by group mean and range scores per criteria dimension. The pre-intervention level of difficulty of attaining respective goals was calculated by mean and range scores. The retrospective level of performance ability scores are calculated as mean and range. The blinded assessor of GAS guessed the group condition of each participant at pre-and post-data collection to assess whether blinding was maintained. The proportion of correct guesses at pre-and post-intervention is reported. Finally, between-group description of side effects by NEQ is calculated by frequency of total endorsed items, and the average impact of each reported side-effects is calculated from the NEQ.

## Results

### Attendance and completion

The intervention completion rate for SKOLKONTAKT™ was 70.6% (*n* = 12) and 87.5% (*n* = 14) for social activity controls. About one third, 33% (*n* = 4; range 35–100% session attendance), of SKOLKONTAKT™ completers and 36% (*n* = 5; range 18–100% session attendance) social activity group completers attended 80% or more of intervention sessions. During the study, five participants dropped out from the SKOLKONTAKT™ groups (3 before intervention week 2). They reported rationales for dropping out which were not clearly related to the intervention itself, including overall low school attendance and motivation, general anxiety, and demands to transition to remote sessions. Similarly, overall low school attendance and general anxiety was reported as rationales for the two dropouts in the social activity control groups. See Table 1 for more information on dropouts.

Reported and unreported school absence was significantly higher in the control groups in comparison to the SKOLKONTAKT™ groups for the duration of the parallel intervention (F1/24 > 6.2, *p* < 0.01, *ɳ*^2^ = 0.09). School attendance and session attendance in SKOLKONTAKT™ was positively correlated (*r* = 0.75, *p* < 0.01). School absence and attendance on sessions in SKOLKONTAKT™ was negatively correlated (*r* = −0.43, *p* = 0.02).

### Social communication skills (SSGQ)

SKOLKONTAKT™ had neither a superior multivariate (F1/24 < 3.8, *p* > 0.07; *ɳ*^2^ = 0.03) effect on social activity control nor univariate effects on participant, parent, or teacher ratings for SSGQ total scores at post-intervention or at follow-up (Supplementary Table S3). On the SSGQ item level, SKOLKONTAKT™ groups showed significant improvement over social activity control on several individual items at follow-up by participant report (*t* > 1.8, *p* < 0.04), including “Meeting new people” (Item 1), “Beginning a conversation” (Item 2), “Asking for a favor appropriately” (Item 7), “Seeking help from peers appropriately” (Item 8), “Seeking help from adults appropriately” (Item 9), “Playing a game successfully” (Item 12), and “Accepting a compliment” (Item 18). Moreover, parent ratings showed improvements following SKOLKONTAKT™ over social activity control on several individual items (*t* > 2.1, *p* < 0.02) including “Meeting new people” (Item 1), “Asking for a favor appropriately” (Item 7), “Working cooperatively” (Item 14), and “Demonstrating the ability to understand others’ behavior” (Item 21). Additionally, teachers rated improvements based on SKOLKONTAKT™ for social activity control using two SSGQ items (*t* > 1.9, *p* < 0.04): “Meeting new people” (Item 1) and “Beginning a conversation” (Item 2) (Supplementary Tables S4A–C).

### Quality of life (KIDSCREEN 27)

Significant improvements in overall quality of life were observed for SKOLKONTAKT™ compared to social activity control at follow-up (F1/24 = 3.6, *p* = 0.04; *ɳ*^2^ = 0.04) as well as for the subscales Physical Well-Being (F1/24 = 5.1, *p* = 0.02; *ɳ*^2^ = 0.05) and Peers and Social Support (F1/24 = 4.8, *p* = 0.02; *ɳ*^2^ = 0.04) (Supplementary Table S5).

### Personally meaningful goals (goal attainment scaling)

Quantitative results show that if the goal was formulated as part of SKOLKONTAKT™, a larger proportion of goals, out of total number of goals per group, reached goal-attainment based on the values expected (0), more than expected (+1), or significantly more than expected (+2) than had the goal been formulated as a part of the control groups. Conversely, progress below attainment, i.e., at somewhat less (−1) or significantly less than expected (−2), was more likely had the goal been formulated as a part of the social activity control groups. This suggest that socially important, school-related goals are more likely attained given intervention with SKOLKONTAKT™ than other social activity peer groups. See Table 2 for details.

**Table 2:** GAS scoring outcome overview for goals with most and second to most progress as well as thematic goal-setting areas.

Group	Much less than expected, −2 *n* (% group total)		Somewhat less than expected, −1 *n* (% group total)		Expected outcome, 0 *n*(% group total)		Somewhat more than expected, 1 *n* (% group total)		Much more than expected, 2 *n* (% group total)		Number of GAS within area		Not completed during interventions**		SKOL-KONTAKT™	Active control	SKOL-KONTAKT™	Active control	SKOL-KONTAKT™	Active control	SKOL-KONTAKT™	Active control	SKOL-KONTAKT™	Active control	SKOL-KONTAKT™	Active control	SKOL-KONTAKT™	Active control
Most progress	1 (8%)	4 (31%)	0 (0%)	1 (8%)	3 (25%)	1 (8%)	5 (42%)	5 (38%)	2 (17%)	1 (8%)	12	13	1	1
Second-most progress	2 (33%)	4 (67%)	0 (0%)	0 (0%)	0 (0%)	0 (0%)	2 (33%)	2 (33%)	2 (33%)	0 (0%)	6	7	0	1
Theme 1: Peer relationship at school*	1 (17%)	4 (40%)	0 (0%)	1 (10%)	1 (17%)	1 (10%)	4 (67%)	3 (30%)	0 (0%)	1 (17%)	6	10	0	0
Theme 2: Social skills in the classroom*	1 (17%)	2 (40%)	0 (0%)	0 (0%)	0 (0%)	0 (0%)	2 (33%)	3 (60%)	3 (33%)	0 (0%)	6	5	0	1
Theme 3: Managing demands at school*	1 (20%)	2 (67%)	0 (0%)	0 (0%)	2 (40%)	0 (0%)	1 (20%)	1 (33%)	1 (20%)	0 (0%)	5	3	1	1

*Most and second to most progress goals included. **One GAS was not thematically categorized or completed during interventions. Student changed goal during intervention.

Thematic analysis suggests three goal formulation themes: ‘Social skills in the classroom’, ‘Managing demands at school’, and ‘Peer relationships at school’. Slightly more goals in SKOLKONTAKT™ related to demand management and somewhat fewer to relationships with peers than in social activity control groups. See Supplementary Table S6 in the supplementary materials for example formulations per theme in the study.

Pre-intervention assessments of goal quality show differences in means suggesting slightly better-quality goals if formulated in SKOLKONTAKT™ (range = 0–0.3 difference). Notably, descriptive presentation of GAS quality suggests improved quality of goal formulation using a goal catalogue. Importantly, no goal fell below a rating of 2 using the goal catalogue, which indicates that goal quality increases such that outcomes may be more clearly represented. See Table 3 for details.

**Table 3:** Group-comparison of pre-intervention average quality rating of all formulated goals (GAS) and student-reported goal difficulty with and with available goal-catalogue.

	Equidistance mean (range)		Measurability mean (range)		Unidimensionality mean (range)		Overlapping mean (range)		Relevance mean (range)		Prospective difficulty mean (range)	
	SKOL-KONTAKT™	ACTIVE CONTROL	SKOL-KONTAKT™	Active control	SKOL-KONTAKT™	Active control	SKOL-KONTAKT™	Active control	SKOL-KONTAKT™	Active control	SKOL-KONTAKT™	Active control
All groups	2.4 (1–3)	2.4 (2–3)	2.2 (1–3)	2.4 (1–3)	2.5 (1–3)	2.8 (2–3)	2.1 (1–3)	2.3 (1–3)	2.8 (2–3)	2.9 (2–3)	3 (2–5)	3 (1.5–4)
Groups without goal-catalogue	2.2 (1–3)	2.5 (2–3)	1.7 (1–2)	2.3 (1–3)	2.2 (1–3)	2.7 (2–3)	1.8 (1–2)	2 (1–3)	2.8 (2–3)	2.8 (2–3)	3 (3)	3.2 (2–4)
Groups with goal-catalogue	2.7 (2–3)	2.8 (2.3–3)	2.8 (2.3–3)	2.5 (2–3)	2.9 (2.5–3)	2.9 (2.3–3)	2.5 (2–3)	2.5 (2–3)	2.8 (2–3)	3 (3)	3.2 (2–5)	2.9 (1.5–3.7)

Following-up baseline performance ability retrospectively with group facilitators suggest that the majority of SKOLKONTAKT™ participants could perform social goals included for outcome analysis given adequate support. Social activity control performance abilities varied slightly more. Thus, it is possible that SKOLKONTAKT™ goals may have been more readily attained within the given circumstances of the intervention, such as timeframe and group format. While disagreement in terms of outcome was rare regarding goals in the control groups, it was slightly more frequent for goals in SKOLKONTAKT™. However, the most credible source of outcome was commonly made up of a joint facilitator–student report. See Table 4 for details.

**Table 4:** Group facilitator perceived baseline performance ability, goal outcome disagreement and most credible outcome rater of goals (GAS) included for outcome analysis.

	Retrospective baseline performance ability: *n* (%) participants		Group facilitator-participant outcome disagreement: *n* (%) of goals		Most credible outcome rater: *n* (%) of goals		SKOLKONTAKT™ Missing: 2 (11%)	Active control missing: 3 (14%)	SKOLKONTAKT™ missing: 2 (11%)	Active control missing: 3 (14%)	SKOLKONTAKT™ missing: 1 (6%)	Active control missing: 3 (14%)
*All groups*	At performance: 0 Performance given support: 16 (83%) No performance:1 (6%)	At performance: 1 (5%) Performance given support: 11 (50%) No performance: 6 (27%)	6 (33%)	1 (5%)	Student: 5 (28%) Facilitator: 1 (6%) Both: 11 (61%)	Student: 5 (24%) Facilitator: 2 (10%) Both: 11 (52%)

Finally, pre-to post-intervention assessment of whether the GAS assessor remained blind to what group each participant belonged decreased from 81% correct guesses preintervention to 65% correct guesses postintervention. Arguably, the proportion of correct responses would have been maintained or increased to postintervention had blinding been compromised to the extent that GAS assessment would have been severely affected.

### Side effects

#### Negative Effects Questionnaire

In total, participants mentioned 71 side effect items related to the interventions: 29 in SKOLKONTAKT™ (41%) and 42 endorsed in social activity control groups (59%). Average impact rating of single items in SKOLKONTAKT™ were 0.8 (range = 0–4). One participant indicated low trust in group facilitator with a subjective impact rating of 4 while the remainder impact ratings ranged from 0 to 2 where the impact of reported side effects was low at the group level. The average impact rating in the social activity groups was 0.87 (range = 0–4), which also indicates the low impact of side effects at the group level. No participant in SKOLKONTAKT™ endorsed the item “the intervention does not suit me,” but two participants in the active control condition did.

## Discussion

The aim of the present study was to investigate the acceptability, feasibility, safety, and preliminary efficacy of SSGT SKOLKONTAKT™ for autistic learners and students with formally documented social communication challenges. Despite the COVID-19-related challenges in school and for delivery of the intervention, the majority of SKOLKONTAKT™ participants completed the training. Careful consideration of unwanted effects by group and individual monitoring and measures suggests that the training is a safe with limited or mild/manageable side effects within the school setting. Few studies on SSGT have previously collected data on side effects. However, systematic understanding of side effects may not only optimize intervention safety but also intervention efficacy by the ability to intervene at appropriate times (Afsharnejad et al., 2021). Most dropouts in SKOLKONTAKT™ occurred early in the intervention, which underlines the importance of appropriate *a priori* training inclusion procedures to evaluate among other things students’ motivation and mental health, as indicated by drop-out rationales in the current study. While more participants dropped out from SKOLKONTAKT™ than controls, reasons were comparable and not necessarily related to intervention. In addition, school absence was higher in the control groups than in SKOLKONTAKT™, and school attendance and SKOLKONTAKT™ attendance were positively associated, while school attendance and control intervention were not. SKOLKONTAKT™ facilitated aspects of social communication skills, improved life quality from the primary perspective of students—notably including experiences of peer relationships and social support and physical well-being—in comparison to other social group-based activities with peers.

Moreover, personal social and school-related goals were more likely attained following SKOLKONTAKT™. The present study also clarified relevant themes of goal-formulation using GAS, reflecting students desire to connect with peers, feel included, and participate in classroom education as well as find strategies to manage organizational and routine demands integrated into on-site mainstream schooling—findings supported by qualitative student report elsewhere (Goodall, 2018). Importantly, students’ goals often detail the collaboration and relations between themselves and school staff or other students, such as developing strategies to ask for help in the classroom or to initiate desired peer interactions. This emphasizes the interaction between school accommodations and student functioning in how well the student is adjusted at school (Bölte et al., 2021a). Goal formulations according to GAS may contribute to a sense of self-empowerment in relation to social, desirable goals by operationalizing self-efficacious strategies to attain them. Indeed, qualitative student reports suggest that goal-formulation according to GAS was experienced as one of the most meaningful components of SKOLKONTAKT™ (Leifler et al., 2022b). Indeed, this quantitative study of SKOLKONTAKT™ aligns with our earlier multi-perspective qualitative interview study of SKOLKONTAKT™, which demonstrated that it is perceived being a socially valid intervention strategy in mainstream school-settings (Leifler et al., 2022b). The present study further detailed the development of listed prepared goals and corresponding Goal Attainment Scales for use in SKOLKONTAKT™, i.e., the Goal catalogue. Findings suggest using the Goal catalogue might increase not only the quality of goal-formulations but also reduce time consumption of goal-setting procedures. Demonstrations of acceptability and efficiency in the present study suggest that this tool and these procedures are a feasible further adaptation of KONTAKT™ to SKOLKONTAKT™ and will therefore be retained as part of the SKOLKONTAKT™ manual.

Compared to clinical training with KONTAKT™, findings of this study indicate selected and specific effects on social skills questionnaire items rather than overall improvement of social communication skills. A major difference between KONTAKT™ and SKOLKONTAKT™ is that school staff are in charge of the training, not experienced clinicians, which may impact of social communication outcomes, especially if SKOLKONTAKT™ is administered for the first time. Indeed, adequate training, supervision, and resources (i.e., funding, staff, and preparation) is crucial for successful implementation as evidenced by stakeholder interviews (Leifler et al., 2022b). Another change for students that may have contributed to the found patterns of results in compared with clinical delivery of KONTAKT™, was moving the intervention to an online format following pandemic-related social distancing protocols at school. Like KONTAKT™, SKOLKONTAKT™ activities have two main foci. One focus is on increasing social awareness and problem-solving skills (e.g., by activities like group discussions and homework on functional analysis on social situations), and the other is on direct practice of social skills (e.g., recognition and expression of emotions and other group activities). Perhaps intervention components relating to becoming more socially aware and problem-solving in SKOLKONTAKT™ were less affected by the change to online format than aspects in which participants practice social skills more directly, e.g., using others social behaviors to modulate one’s own in conversations when practicing conversation in one group activity. If so, one might not expect clear effects on general social skills but on abilities to understand and problem-solve unique social concerns, as better reflected in our secondary outcomes goal attainment and quality of life. Indeed, superior effects on quality of life and the attainment of social meaningful goals indicate that SKOLKONTAKT™ has added value, even when contrasted with other social activities of comparable lengths and group size. Critiques of social skills training curricula suggest that such programs may contribute to a sense of inauthenticity by training autistic people in “masking” behaviors (Bottema-Beutel et al., 2018). While the authors of the present study encourage increased attention to further exploring active, effective, and safe components of social skills training curricula, the current findings might also indicate that these fears might be unfounded following SKOLKONTAKT™ intervention. Rather than generic social skills behavior effects, the main effects were on well-being and personal meaningful social outcomes. Indeed, benefits in terms of personalized, socio-communicative goals and reduced anxiety in comparison to active controls have been previously reported for the English adaptation of c-SSGT KONTAKT™, the model of SKOLKONTAKT™, for Australian adolescents (Afsharnejad et al., 2021).

Limitations of the study include the small sample size. Additionally, the exploratory and uncorrected nature of parts of the data analysis increase the likelihood of false positive findings, particularly in the analysis of the primary (although preliminary) outcome measure. While overall effects were statistically insignificant, we found significant improvements for social skills on the item level. While interpretation of these effects should be made with all necessary caution it is noteworthy that there were some similarities between responders, including blinded teacher ratings. This might lend support to true, common effects of SKOLKONTAKT™ on single aspects of socio-communicative skills. Moreover, SKOLKONTAKT™ may not be readily available in international contexts as is. The needs-based, rather than diagnosis-based, system of providing educational support in Swedish schools requires careful consideration of socially valid inclusion criteria of students in SKOLKONTAKT™. Among other cultural factors to be considered, inclusion criteria might need further specification in any international use of SKOLKONTAKT™. While scarce in SSGT literature generally (Gates et al., 2017), moderator analysis participant characteristics predicting outcomes may further elucidate inclusion criteria and guide facilitators of the program.

In conclusion, SKOLKONTAKT™ was feasible and safe in an inclusive educational setting in Sweden. We identified specific associations with goal-attainment and student-perceived quality of life in comparison to social activity controls. While the present study could not provide support for the main effects on primary outcome measures, specific aspects of common socio-communicative skills may improve following SKOLKONTAKT™. Future large-scale studies may investigate the adequate primary outcomes of SKOLKONTAKT™, focusing on the social problem-solving and self-empowerment of autistic and other learners requiring social support at school.

## Data availability statement

The datasets presented in this article are not readily available because Raw data cannot be made immediately publicly available, as this was not specified in ethical approval, and parts of it (e.g., Goal Attainment Scale), are not completely anonymous. Thus, raw data can currently only be provided by the authors upon legitimate request and additional procedures (e.g., ethical amendment; additional anonymization of verbal data). The authors can provide without delay additional details on the data in processed form. Requests to access the datasets should be directed to anna.fridell@ki.se.

## Ethics statement

The studies involving human participants were reviewed and approved by Swedish Ethical Review Board. Written informed consent to participate in this study was provided by the participants’ legal guardian/next of kin.

## Author contributions

AF and SB made contributions toward the scope, structure, methods, and key findings of this research manuscript and contributed to the formal descriptive analysis, tables, methodology, writing—original draft preparation, and writing—review and editing. AF, CC, AB, and SB made contributions of the content and structure of the social skills group training and contributed to the conceptualization. AF, CC, and AB were active in the pilot and randomized controlled trial. AF and CC contributed to the data curation. CC contributed to the randomization. AF contributed to the blinding and goal assessment. SB contributed to the supervision and formal inferential analysis. CC, AF, and SB contributed to the validation. All authors contributed to the article and approved the submitted version.

## Funding

This study was supported by Marcus and Amalia Wallenbergs Minnesfond.

## Conflict of interest

SB receives royalties from Hogrefe publishers for KONTAKT™ materials.

The remaining authors declare that the research was conducted in the absence of any commercial or financial relationships that could be construed as a potential conflict of interest.

## Publisher’s note

All claims expressed in this article are solely those of the authors and do not necessarily represent those of their affiliated organizations, or those of the publisher, the editors and the reviewers. Any product that may be evaluated in this article, or claim that may be made by its manufacturer, is not guaranteed or endorsed by the publisher.

## Footnotes

^1^randomization.org
