# Supplementary material for: School-based social skills group training (SKOLKONTAKT™): a pilot randomized controlled trial
Source: Front Psychol. 2023 Jul 27;14:1128288. doi: 10.3389/fpsyg.2023.1128288 (PMC10412818; doi:10.3389/fpsyg.2023.1128288)
Supplement: Supplementary file 1 [file Data_Sheet_1.PDF]

Supplementary materials

Table S1: Main activity and content of each session in SKOLKONTAKT™.

	**SKOLKONTAKT™ participant group**			**Parents and other school staff**
	Session 1 (50 min)<br>Mission and Snack time	Session 1 (50 min)<br>Theme-based discussion	Session 1 (50 min)<br>Group activity	Weekly activities
**Week 1:**	Introduction to SKOLKONTAKT™ structure and content.<br><br>Presenting goals, missions and activity book.	Presenting yourself in group-settings. Discuss why social situations may become difficult using cognitive models.	Social initiatives to peers during a structured game-setting.	Weekly information on SKOLKONTAKT™ content in activity book.
**Mission:**	Formulating social goal AND identifying supporters to help during training.			
**Week 2:**	Missions: Evaluating previous and introducing new mission.<br><br>Snack time: Trainer-guided, unstructured social interaction during snack time.	Being part of a group.	Formulating group rules.	Weekly information on SKOLKONTAKT™ content in activity book.
**Mission:**	Functional analysis of goal-related situation OR registration of goal-related situations.			
**Week 3:**	Missions<br>Snack time	What are and how do you talk about your social strengths and challenges.	Cooperative group game.	Weekly information on SKOLKONTAKT™ content in activity book.
**Mission:**	Functional analysis of goal-related situation			
**Week 4:**	Missions<br>Snack time	Understanding non-verbal communication of emotions	Recognizing non-verbal communication using a computerized material.	Weekly information on SKOLKONTAKT™ content in activity book.
**Mission:**	Functional analysis of situation involving interpretation of non-verbal communication.			
**Week 5:**	Missions<br>Snack time	Managing misunderstandings.	Discussing non-verbal communication while watching a video.	Weekly information on SKOLKONTAKT™ content in activity book.
**Mission:**	Functional analysis of situation involving using an alternative behavior to resolve a social situation.			

**Week 6:**	Missions<br>Snack time	Social initiatives	Practice initiatives with someone not part of the training-group.	Weekly information on SKOLKONTAKT™ content in activity book.
**Mission:**	Functional analysis of situation involving taking a social initiative.			
**Week 7:**	Missions<br>Snack time	Managing (feelings of) social exclusion.	Role play.	Weekly information on SKOLKONTAKT™ content in activity book.
**Mission:**	Functional analysis of situation feeling excluded socially.			
**Week 8:**	Missions<br>Snack time	Managing difficult social situations at school.	Charades.	Weekly information on SKOLKONTAKT™ content in activity book.
**Mission:**	Functional analysis of situation involving a difficult social situation at school.			
**Week 9:**	Missions<br>Snack time	Socializing outside known environments (joint excursion).	Role play.	Weekly information on SKOLKONTAKT™ content in activity book.
**Mission:**	Preparing a practice activity for joint excursion. Functional analysis of own practice during the joint excursion.			
**Week 10:**	Missions<br>Snack time	Managing environmental changes when socializing.	Recognizing non-verbal communication using a computerized material.	Weekly information on SKOLKONTAKT™ content in activity book.
**Mission:**	Functional analysis of situation involving a sudden change.			
**Week 11:**	Missions<br>Snack time	Entering young adulthood.	Discussion ambiguous social situations using a validated digital material.	Weekly information on SKOLKONTAKT™ content in activity book.
**Mission:**	Evaluating goal setting. Identifying maintenance strategies.			
**Week 12:**	Missions<br>Snack time	Evaluation and maintenance strategies following SKOLKONTAKT™.	Optional activity.	Weekly information on SKOLKONTAKT™ content in activity book.

Note: Adapted overview from SKOLKONTAKT™ manual. Each week is conducted over three sessions. Each session includes opening and closing rounds as well as intro/evaluation or coaching of weekly mission in a recurring structure. Parents and other school staff (e.g., student mentors) receive written information only.

Table S2: Overview of SKOLKONTAKT™ intrinsic principles of training and examples of how they are using in session activities and trainer approach.

Principle	Session activities	Trainer approach
**Clear structure and communication**	Recurring agenda	Minimizing but preparing any changes to program structure.
**Agreements**	Group rules	Providing clear session structure while welcoming ideas and suggestions from participants. Using problem-solving approach to agreeing on decisions as a group.
**Supportive and constructive feedback**	All activities	Positive reinforcement, clarify social expectations, problem-solving and antecedent analysis of social situations, clarifying alternative social behaviors when necessary.
**Repetition**	Recurring agenda	Reiterating ideas and materials from previous sessions. Summarizing discussions.
**Goal-based practice**	Group- and individualized	Clarifying how activities may relate to goals of the group and individual throughout.
**Increasing difficulty**	Incremental introduction of new activities. Training focus on group identity and structure initially and practice increasingly complex skills/situations.	Added focus on new material and topics when necessary.
**Practice of structured and unstructured social communication**	Majority of activities allow clearer structure for social interaction. "Snack time" allow (guided) unstructured interaction, including small talk.	Trainer may suggest topics, questions, or other phrases to use during more unstructured portions of sessions.
**Managing challenging behaviors**	Throughout training. Reiterating group rules. Antecedent-analysis of group rules. Use of social stories.	Use of SKOLKONTAKT™ principles and analyzing trainer behaviors to prevent challenging behaviors.
**Use of helpful tools**	Visual aid and tools that are part of the program, including visualization of emotional states, antecedent-analysis problem-solving. Also, social stories, cognitive aids, and individual meetings and other support when necessary.	Continuous assessment of students' engagement and understanding during the sessions. Thoughtful and motivated introduction of support and aids whenever necessary.
**Strengths and interests**	Throughout training.	Use of student interests and strength during discussions and activities to motivate, reinforce and rationalize training.

Table S3: Pre. post and follow-up SSGQ total scores per intervention group and informant type.

	PRE-INTERVENTION MEAN (SD)		POST-INTERVENTION MEAN (SD)		FOLLOW-UP MEAN (SD)		
	SKOLKONTAKT	ACTIVE CONTROL	SKOLKONTAKT	ACTIVE CONTROL	SKOLKONTAKT	ACTIVE CONTROL	**F (p);** η^2
**STUDENT**	75.21 (22.15)	76.50 (17.05)	78.71 (12.89)	76.17 (17.15)	79.43 (13.52)	76.67 (20.27)	.35 (.56); .01
**PARENT**	74.71 (18.45)	75.25 (12.27)	76.71 (10.72)	73.42 (16.79)	78.93 (14.89)	72.50 (17.93)	.99 (.33); .04
**TEACHER**	65.00 (16.30)	68.75 (22.19)	69.14 (14.16)	70.08 (21.87)	69.79 (11.40)	70.17 (22.73)	3.8 (.06); .14

Table S4A: <u>Student reported</u> SSGQ item score pre-post in SKOLKONTAKT vs social activity

	**PRE-INTERVENTION MEAN (SD)**		**FOLLOW-UP MEAN (SD)**		
	SKOLKONTAKT	ACTIVE CONTROL	SKOLKONTAKT	ACTIVE CONTROL	t (p)
**ITEM 1**	2.08 (0.67)	2.43 (0.85)	2.50 (0.80)	2.63 (0.73)	1.8 (.04)
**ITEM 2**	2.33 (0.98)	2.07 (0.83)	2.75 (1.06)	2.23 (1.16)	1.8 (.04)
**ITEM 3**	4.00 (0.74)	3.86 (1.29)	4.17 (1.03)	3.64 (0.63)	0.6 (.57)
**ITEM 4**	3.08 (1.16)	2.86 (0.95)	3.25 (1.29)	3.07 (0.73)	0.5 (.64)
**ITEM 5**	2.50 (1.00)	2.71 (0.99)	2.83 (1.59)	2.64 (0.74)	1.3 (.22)
**ITEM 6**	3.42 (0.67)	3.50 (1.22)	3.83 (1.03)	3.50 (0.85)	1.6 (.14)
**ITEM 7**	3.17 (1.19)	3.21 (1.12)	3.83 (1.03)	3.21 (1.05)	2.2 (.02)
**ITEM 8**	2.42 (1.31)	3.00 (1.57)	3.25 (1.48)	3.00 (0.96)	3.1 (.005)
**ITEM 9**	3.67 (1.50)	3.36 (1.55)	4.17 (1.19)	3.43 (0.94)	1.9 (.03)
**ITEM 10**	3.92 (0.79)	4.07 (1.33)	4.17 (1.11)	3.93 (1.07)	1.3 (.21)
**ITEM 11**	3.58 (1.00)	2.93 (1.21)	3.58 (1.08)	3.00 (0.96)	1.5 (.15)
**ITEM 12**	3.33 (1.15)	3.50 (1.45)	3.92 (1.38)	3.79 (0.80)	1.9 (.04)
**ITEM 13**	2.83 (1.34)	2.71 (1.54)	3.50 (1.51)	2.64 (0.93)	0.2 (.84)

**ITEM 14**	3.67 (1.30)	3.57 (1.50)	3.75 (1.48)	3.50 (1.09)	0.7 (.50)
**ITEM 15**	3.75 (1.14)	3.43 (1.34)	3.92 (1.24)	3.14 (1.41)	0.5 (.62)
**ITEM 16**	4.58 (1.00)	4.36 (1.45)	4.75 (0.97)	4.07 (1.07)	0.8 (.43)
**ITEM 17**	3.75 (1.06)	3.36 (1.78)	3.92 (1.08)	3.21 (1.42)	0.6 (.55)
**ITEM 18**	2.50 (1.38)	3.00 (0.88)	2.80 (0.95)	2.79 (0.97)	1.9 (.04)
**ITEM 19**	4.17 (1.19)	3.57 (1.60)	3.92 (1.08)	3.63 (0.92)	0.6 (.56)
**ITEM 20**	4.25 (1.36)	3.50 (1.74)	3.83 (1.53)	3.57 (0.76)	1.2 (.27)
**ITEM 21**	3.58 (1.38)	3.21 (1.42)	3.75 (1.48)	3.14 (0.86)	0.4 (.67)
**ITEM 22**	2.50 (1.00)	2.86 (1.35)	3.08 (1.38)	3.07 (1.14)	1.6 (.13)
**ITEM 23**	3.42 (1.24)	4.14 (1.56)	3.00 (0.95)	3.79 (1.31)	1.6 (.14)

Table S4B: **<u>Parent reported</u> SSGQ item score pre-post in SKOLKONTAKT vs social activity**

	**PRE-INTERVENTION MEAN (SD)**		**FOLLOW-UP MEAN (SD)**		
	SKOLKONTAKT	ACTIVE CONTROL	SKOLKONTAKT	ACTIVE CONTROL	t (p)
**ITEM 1**	2.08 (0.67)	2.29 (0.91)	2.75 (0.97)	2.36 (0.74)	2.1 (.02)
**ITEM 2**	2.17 (0.72)	2.00 (0.96)	2.67 (0.89)	2.64 (1.08)	1.6 (.14)
**ITEM 3**	2.92 (1.00)	3.71 (1.27)	3.17 (1.03)	3.79 (1.31)	0.9 (.39)
**ITEM 4**	2.83 (0.94)	3.14 (1.10)	3.08 (0.90)	3.21 (1.25)	0.8 (.46)
**ITEM 5**	2.25 (0.75)	2.07 (0.92)	2.50 (0.80)	2.50 (0.94)	0.7 (.54)
**ITEM 6**	3.17 (0.83)	3.36 (1.15)	3.58 (1.00)	3.57 (0.85)	1.2 (.27)
**ITEM 7**	3.08 (0.90)	3.71 (1.20)	3.75 (1.22)	3.43 (0.85)	2.6 (.005)
**ITEM 8**	2.17 (1.11)	3.21 (1.31)	2.75 (1.22)	3.14 (1.03)	1.5 (.15)
**ITEM 9**	3.17 (1.11)	3.29 (1.07)	3.50 (1.31)	3.43 (0.85)	1.0 (.34)
**ITEM 10**	3.50 (0.67)	3.79 (1.05)	3.67 (1.23)	4.00 (0.68)	0.4 (.67)
**ITEM 11**	3.00 (1.41)	3.43 (1.40)	2.67 (1.07)	3.21 (1.42)	0.9 (.39)
**ITEM 12**	2.92 (0.79)	3.57 (1.22)	3.33 (1.07)	3.29 (0.91)	1.6 (.14)
**ITEM 13**	2.50 (0.80)	2.29 (1.14)	3.08 (1.38)	2.49 (0.80)	1.5 (.17)

**ITEM 14**	2.58 (0.90)	3.21 (1.19)	3.33 (1.23)	3.43 (1.02)	2.2 (.03)
**ITEM 15**	3.08 (1.00)	3.71 (1.20)	3.17 (1.34)	3.57 (1.28)	0.2 (.82)
**ITEM 16**	3.58 (1.56)	4.07 (1.33)	3.83 (1.40)	4.14 (1.23)	0.9 (.39)
**ITEM 17**	3.17 (1.19)	3.14 (1.23)	3.42 (1.51)	3.36 (0.93)	0.9 (.39)
**ITEM 18**	3.00 (0.85)	3.21 (1.19)	3.33 (1.07)	3.07 (1.07)	1.3 (.22)
**ITEM 19**	2.83 (1.03)	3.86 (1.29)	2.92 (1.08)	3.79 (0.97)	0.4 (.72)
**ITEM 20**	2.75 (1.14)	3.57 (1.16)	2.67 (1.15)	3.50 (1.45)	0.4 (.73)
**ITEM 21**	2.58 (1.03)	3.50 (1.16)	3.17 (1.16)	3.21 (1.19)	3.0 (.005)
**ITEM 22**	2.83 (0.67)	3.00 (0.88)	3.08 (0.83)	3.29 (1.07)	1.1 (.28)
**ITEM 23**	3.08 (1.16)	3.57 (0.94)	3.08 (1.24)	3.21 (1.19)	1.2 (.25)

Table S4C: Teacher reported SSGQ item score pre-post in SKOLKONTAKT vs social activity

	PRE-INTERVENTION MEAN (SD)		FOLLOW-UP MEAN (SD)		
	SKOLKONTAKT	ACTIVE CONTROL	SKOLKONTAKT	ACTIVE CONTROL	t (p)
**ITEM 1**	2.17 (0.72)	2.14 (0.95)	3.00 (1.21)	2.07 (0.83)	2.4 (.008)
**ITEM 2**	2.50 (1.09)	2.29 (1.20)	3.08 (1.24)	2.44 (0.93)	1.9 (.04)
**ITEM 3**	3.58 (1.31)	3.50 (0.65)	3.50 (0.80)	3.21 (0.70)	0.2 (.83)
**ITEM 4**	2.83 (1.03)	2.64 (0.93)	2.83 (0.94)	2.64 (0.63)	0.4 (.65)
**ITEM 5**	3.50 (1.57)	2.86 (1.17)	3.25 (1.14)	2.29 (0.73)	1.4 (.19)
**ITEM 6**	3.42 (1.31)	3.29 (1.07)	3.67 (1.15)	3.14 (0.86)	0.6 (.59)
**ITEM 7**	3.08 (1.16)	3.00 (0.96)	3.42 (1.38)	2.71 (0.91)	0.7 (.49)
**ITEM 8**	2.50 (1.09)	2.14 (1.03)	2.92 (1.08)	1.86 (0.77)	1.2 (.27)
**ITEM 9**	3.00 (1.21)	2.71 (0.99)	3.58 (1.31)	2.86 (0.77)	1.4 (.19)
**ITEM 10**	3.00 (0.74)	2.79 (0.70)	3.42 (1.16)	2.57 (0.65)	1.3 (.21)
**ITEM 11**	2.92 (0.79)	2.50 (0.85)	3.33 (0.98)	2.57 (0.65)	1.3 (.21)
**ITEM 12**	3.00 (1.13)	2.43 (0.94)	2.92 (1.38)	2.36 (0.63)	0.2 (.82)

**ITEM 13**	2.50 (1.31)	1.86 (0.77)	2.67 (1.37)	1.71 (0.73)	0.4 (.72)
**ITEM 14**	3.50 (1.31)	2.79 (0.80)	3.08 (1.16)	2.50 (0.65)	0.9 (.39)
**ITEM 15**	2.67 (1.07)	2.36 (1.08)	2.92 (1.24)	2.07 (0.47)	0.6 (.58)
**ITEM 16**	3.33 (1.15)	3.14 (0.86)	3.58 (1.24)	2.79 (0.58)	0.5 (.59)
**ITEM 17**	2.58 (1.24)	2.00 (0.68)	2.50 (1.31)	2.07 (0.73)	0.2 (.85)
**ITEM 18**	3.00 (0.74)	2.43 (0.85)	2.92 (1.00)	2.43 (0.76)	0.4 (.72)
**ITEM 19**	3.00 (0.95)	2.57 (0.85)	3.33 (1.23)	2.64 (0.74)	1.1 (.31)
**ITEM 20**	2.92 (1.31)	2.43 (1.09)	3.17 (1.03)	2.36 (0.74)	0.6 (.54)
**ITEM 21**	3.08 (1.31)	2.50 (1.22)	3.25 (1.06)	2.36 (0.74)	0.4 (.64)
**ITEM 22**	2.50 (1.09)	2.21 (0.80)	2.58 (1.44)	1.86 (0.53)	0.3 (.80)
**ITEM 23**	4.17 (1.80)	3.43 (1.16)	3.75 (1.29)	3.07 (1.00)	1.5 (.18)

Table S5: Pre. post and follow-up KIDSCREEN-27 total and index scores per intervention group and informant type.

	**PRE-INTERVENTION MEAN (SD)**		**POST-INTERVENTION MEAN (SD)**		**FOLLOW-UP MEAN (SD)**		
	SKOLKONTAKT	ACTIVE CONTROL	SKOLKONTAKT	ACTIVE CONTROL	SKOLKONTAKT	ACTIVE CONTROL	F(p); η^2
**1. PHYSICAL WELL-BEING**	12.91 (3.45)	15.40 (3.86)	14.91 (2.81)	14.60 (4.65)	15.27 (3.20)	14.20 (4.69)	5.1 (.02); .05
**2. PSYCHOLOGICAL WELL-BEING**	24.45 (4.23)	25.00 (5.79)	25.09 (3.94)	24.60 (4.81)	22.00 (1.73)	22.60 (2.17)	0.5 (.52); .01
**3. AUTONOMY AND PARENTS**	28.09 (3.67)	27.30 (4.11)	28.45 (3.70)	27.40 (4.60)	27.64 (3.26)	28.70 (4.03)	1.5 (.24); 02
**4. PEERS AND SOCIAL SUPPORT**	9.18 (3.84)	14.10 (4.43)	11.73 (4.88)	12.80 (3.49)	10.45 (5.92)	13.40 (3.60)	4.8 (.02); .04
**5. SCHOOL ENVIRONMENT**	15.82 (2.14)	15.60 (2.55)	15.09 (1.92)	14.60 (3.31)	15.27 (1.55)	14.80 (2.82)	0.4 (.54); .01
**TOTAL**	90.45 (12.12)	97.40 (17.34)	95.27 (9.96)	94.00 (13.70)	95.64 (9.42)	93.70 (11.02)	3.6 (.04); .04

Table S6: Examples of goal formulations according to GAS by students across the study (with varying ratings of quality)

Theme	*Much less than expected (-2)*	*Somewhat less than expected (-1)*	*Expected outcome (0)*	*Somewhat more than expected (+1)*	*Much more than expected (+1)*
Theme 1: Peer relationships at school	I think it is difficult to enter ongoing group-discussions	With aid, I have written down at least three strategies I can use to enter an ongoing group discussion.	I have entered an ongoing group discussion during breaktime or on school hours on one occasion by using these strategies.	I have entered an ongoing group discussion during breaktime or on school hours on two occasions.	I have entered an ongoing group discussion during breaktime or on school hours on three occasions.
Theme 1: Peer relationships at school	Difficult to talk to peers, starting and maintain conversations	I have started one conversation and posed a follow-up question on three occasions.	I have started one conversation and posed a follow-up question on at least five occasions.	I have started and maintained the conversation and felt pleased.	I have entered one conversation I wanted to be part of even though it includes several people and some I don’t know well.
Theme 2: Social skills in the classroom.	I ask for help about 2-3 times per school week when I don’t know what to do in the classroom/during breaktime.	With help from my group trainer, I have written down three strategies I can use to ask for help in the classroom. Use cards, raising my hand.	I have on one occasion used one or several of these strategies using various objects etc.	I have on two occasions used one or several of these strategies. Using support words, prepared objects etc. Outside the classroom, during breaks.	I have on two occasions used one or several of these strategies using support words, prepared tips and objects during classes or between classes.
Theme 2: Social skills in the classroom.	In activities at school where we conduct oral presentations in front of the class, I provide written or one-on-one oral presentations instead.	With the help of a group trainer, I have written down three tips/strategies I can use to present something in front of others.	With support from my group trainer, I have used at least one of these strategies to present something to my group.	Without support from my group trainer, I have used at least one of these strategies to present something to my group.	Without support from my group trainer, I have used at least one of these strategies to present something on one occasion to my class.
Theme 3: Managing demands at school	I can come to school and enter the classroom once a week without support.	I can enter the classroom twice a week with support.	I can enter the classroom three times a week with support.	I can enter the classroom four times a week with support.	I can enter the classroom five times a week with support.
Theme 3: Managing demands at school	I find it difficult to occupy myself in between classes, difficult to find something to do.	With help from my Social Activity group leader, I have gotten suggestions and written down appropriate activities.	I have conducted one or two of these suggestions on one occasion.	I have conducted one or two of these suggestions on two occasions.	I have conducted one or two of these suggestions on three occasions.

Note: Free translation to English by author AF.
